# Should Spendthrifts Be Placed under Restraint

**Published:** 1907-09-28

**Authors:** 


### SHOULD SPENDTHRIFTS BE PLACED UNDER RESTRAINT.

From the stage of utter mental darkness we find gradations through all the various phases of insanity up to those forms of mental aberration which are on the very threshold of insanity. Attempts have been made to frame a general definition of all these phases, but without success. They run, like the gradations of colour, so much into one another that those contiguous to each other seem the same, whilst those more distant seem to have no relation whatever for the purposes of a definition, their differences being so great. The divisions of idiocy and lunacy, monomania and imbecility adopted by jurists, point to well-marked phases of the disease, but leave those others untouched which are intermediate, and hence in former times arose a legal difficulty in deciding whether he was an idiot or a lunatic, who was something between both.

The Roman Law dealt with prodigals and spendthrifts in much the same manner as lunatics. Speaking of such characters, Ulpian, the great Roman lawyer, says: "By the Law of the Twelve Tables a Prodigus was interdicted from the administration of his property. This rule of law was first based upon legal custom, but nowadays, prætors or præsides, finding such a man who puts no limit to the direction or amount of his expenses, but exhausts his substance in a destructive and dissolute manner, are wont to give him a Curator after the example of a Furiosus [madman], and both of them are confined under the guardianship of the curator until in the case of the furiosus he recovers from his insanity, and of the prodigus, he returns to good behaviour, which event, when it happens, absolves them respectively from the guardianship of their curators." The legal incapacity of a prodigal to act was founded upon a similar reason to that which denied a like capacity to minors and women—namely, the supposition of a mental infirmity. The deprivation of his capacity to act assumes that the prodigus was previously possessed of that capacity, and that he was not labouring under the incapacity of age. The words of the decree made in such cases were as follows: "Inasmuch as your ancestral property left to you is squandered by you iniquitously, and your children reduced to poverty, I for such cause interdict you the administration of the same, and also from all commercial dealing with others." He could not alone alienate his property, nor could he make a money payment, and if he did his curator could recover it back. His prodigality was obliged to be proved from acts, not words. Nor was the law against prodigality confined to the male sex, for women of extravagant habits, the "pretty horse-breakers," and costly dressing damsels of ancient Rome were fit subjects of the interdict. But in addition to being deprived of their legal capacity, the prodigi were subjected to a species of infamia similar to that which prevailed in the Greek laws of Solon, whereby they were excluded from all public offices. By the Lex Roscia, prodigi were not allowed to sit within the fourteen rows in the theatre but were allotted distinct places. Many senators were removed from the senate and degraded, some by the censors and some by the Emperors, for prodigality, particularly by Tiberius Cæsar, as mentioned by Tacitus. Adrian used to have them brought into the forum and publicly disgraced. Some were kept in durance vile in their family vaults as a sort of black hole; some were fined and punished in various ways.

The Roman law of prodigals did not continue to preserve its stringent character. It was greatly modified by the Emperor Leo (Const. 39). This constitution was enacted to remedy the abuses which had arisen from a maladministration of the law, and, after stating that no man was so perfect in his conduct as not to act foolishly sometimes, nor any man so imprudent but what he might occasionally act discreetly, adjudged that whilst a prodigal was under the *interdictio bonorum*, such of his acts as were acts of discretion should have validity—otherwise not so. The result of this enactment was to introduce an amount of uncertainty as to acts of prodigality, which did not previously exist. The question, then, necessarily arose whether such acts were of that character as to exempt them from the operation of the interdiction. The evil of this uncertainty was greater than the evil it sought to remedy. But the same difficulty which pressed upon the Emperor Leo afterwards pressed upon the framers of the Code Napoleon.

The above is a concise view of the leading features of the Roman law of Insanity and Prodigality. The Romans were not only a warlike, but also a very practical, common-sense people. They legislated much and well. The tenacity with which the Roman law held its ground in different parts of Europe after the destruction of the empire was not due to its Roman birth, but to its intrinsic excellence being suitable to the wants of men, apart from their nationalities. Although the varying conditions of different States necessitated modifications of that law, yet in its leading principles it remained the same. The law as to furiosi being incapable would be pretty much the same in all nations, the incapacity being founded on a natural fact (mental incapacity), about which all reasonable men must entertain a similarity of opinion. With prodigals it is different; nations may well disagree as to what is, or is not, an act of prodigality, some having more stringent notions upon it than others. Then, again, there may be a diversity of opinion as to the expediency of legally restraining prodigality, some thinking it better that a subject should have the power of beggaring himself and his family, others the contrary. England and America have adhered to the first view—most Continental nations to the second.

We now come to the English law upon insanity and prodigality. With the exception of the sumptuary laws, and a passage in Horne's "Mirror of Justice," the English system did not restrain prodigality. Lord Hardwicke, in *Barnsley, Exparte*, said, "Possibly the law may be too strict, and it might be useful in some cases that a curator should be set over prodigal and weak persons, as in the Roman law." Lord Eldon, in *Ridgway* v. *Darwin*, "felt a strong inclination that the Legislature should take measures to preserve persons in a state of imbecility, laying them open to as much mischief as insanity." Lord Thurlow expressed a similar opinion. As a rule, it is the families, and not the representatives of them, which make a nation great; and in matters of legislation the interest of the family rather than the licentiousness of the representatives, should be considered. But it may be objected that legal restraint of prodigality would only have the effect of saving the *debris* of the property. Scarcely so; for the knowledge that he could be restrained by law would make the prodigal more circumspect in his behaviour, the opportunity for dissipation would be lessened, and the inducement likewise. The mere prospect of saving only the wreck of a noble estate should no more prevent the law from withholding assistance than the prospect of saving only a small portion of burning property should prevent the firemen from exerting themselves to put out the fire. Sir Thomas Ridley complained that a prodigal "is suffered to waste and spend his goods until there be nothing left (as though the prince and the commonwealth had no interest in such a subject, to see he did not waste his estate and abuse his goods), whereby many great houses are overthrown, and many children whom the fathers carefully provided for, never leaving raking and scraping all their lifetime that their children after them might live in great plenty and abundance, come to great shame and beggary." It is a difficult matter to say how far the laws should interfere with a man in the disposition by him of his property; but the same discrimination which can determine what is an act of wanton cruelty to an animal by its owner is surely able to decide what is an act of wanton prodigality of an estate by its owner. Had the principle of the Roman law been bad it is scarcely likely that it would have held its ground from one end of the Continent of Europe to the other for so many centuries; and to suppose that all these civilised nations are wrong in their legislative notions upon this point, and that England alone is right, recalls to mind the expression of Cicero ("De Orat." I. 44)—*Incredibile est enim quam sit omne jus civile praeter hoc nostrum inconditum et paene ridiculum.*

